# Non-linear association between daily mean temperature and children’s hand foot and mouth disease in Chongqing, China

**DOI:** 10.1038/s41598-023-47858-3

**Published:** 2023-11-21

**Authors:** Lin Yang, Tian Liu, Dechao Tian, Han Zhao, Yu Xia, Ju Wang, Tingting Li, Qin Li, Li Qi

**Affiliations:** 1 Chongqing Municipal Center for Disease Control and Prevention, Chongqing, 400042 China; 2 Jingzhou Center for Disease Control and Prevention, Hubei, 434000 China; 3 School of Public Health (Shenzhen), Shenzhen Campus of Sun Yat-Sen University, Shenzhen, 518107 Guangdong China

**Keywords:** Climate sciences, Diseases, Risk factors

## Abstract

Chongqing was seriously affected by hand, foot, and mouth disease (HFMD), but the relationships between daily mean temperature and the incidence of HFMD remain unclear. This study used distributed lag nonlinear model to evaluate the effect of daily mean temperature on the incidence of HFMD in children aged < 5 years in Chongqing. Daily HFMD data from 2012 to 2019 in Chongqing were retrieved from the notifiable infectious disease surveillance system. A total of 413,476 HFMD cases aged < 5 years were reported in Chongqing from 2012 to 2019. The exposure–response curve of daily mean temperature and daily HFMD cases was wavy-shaped. The relative risks (RRs) increased as daily mean temperature below 5.66 °C or above 9.43 °C, with two peaks at 16.10 °C and 26.68 °C. The RRs reached the highest when the daily mean temperature at 26.68 °C on the current day (RR = 1.20, 95% CI 1.09–1.32), followed by the daily mean temperature at 16.10 °C at lag 5 days (RR = 1.07, 95% CI 1.05–1.08). The RRs for girls and daycare children were much higher than those for boys and scattered children, respectively. Taken together, daily mean temperature has strong effect on HFMD in children aged < 5 years old in Chongqing, particularly for girls and daycare children.

## Introduction

Hand, foot, and mouth disease (HFMD) is a common contagious viral disease in children and mainly occurs in East and Southeast Asia including China, which is caused by a group of enterovirus^1,2^. Most HFMD cases are mild and self-limited; however, some cases rapidly develop serious complications such as aseptic meningitis, myocarditis, pulmonary edema, and even death^3,4^. HFMD has been prevalent in China since 2008. A total of 13 million HFMD cases were reported from 2008 to 2015 in China, including 123,261 severe cases and 3322 deaths^5^. Although enterovirus 71 (EV71) vaccines were put into the market in China to prevent EV71-associated diseases in 2017, there was still a lack of effective measures to prevent HFMD caused by other enteroviruses^6,7^. According to the Health Statistic in China, the annual incidence of HFMD was 54.32–178.16 per 100,000, and 348 deaths were reported between 2016 and 2020 (http://www.nhc.gov.cn/). Therefore, it is important to identify risk factors of HFMD and take effective measures to mitigate their effects.

Previous studies have shown that HFMD is affected by many factors, such as meteorological factors, immunity of vulnerable groups, vaccination, and implementation of public health measures^8^. Meteorological factors are one of the leading risk factors. Previous studies indicated that temperature, sunshine, wind speed, relative humidity, and rainfall might be related to the incidence of HFMD^9–12^. Several studies showed that the daily mean temperature played the greatest role among meteorological factors^13–16^. Temperature influences the survival and spread of infectious pathogens in the environment, as well as the behaviors and activities of the population, thereby influencing the dynamics of infection transmission^17^. Warmer temperatures accelerate viral reproduction and prolong viral survival, concurrently changing people's behavioral and activities, including eating habits and lifestyles, thereby increasing the risk of exposure and infection. Previous studies showed that the association between temperature and HFMD varied across regions^14,18–20^. For example, some studies in mainland China^21^ showed a non-linear exposure–response relationship with an approximating inverted V-shape, while some studies in eastern China found an approximate M-shaped relationship^13,22^. Besides, studies in Singapore and Japan showed a threshold and J-shaped and an inverted V-shaped relationship, respectively^23,24^. Therefore, it is critical to assess the response of HFMD to daily mean temperature on a local basis.

Chongqing, the largest municipality, is located in the southwestern of mainland China and was seriously affected by HFMD. The HFMD incidence in this region was much higher than the national incidence and that of many countries or regions^8^. The study of Qi et al. showed that a total of 276,207 HFMD cases were reported in Chongqing during 2009–2016, with 91.3% of cases occurring in children under 5 years old^25^. The relationship between daily mean temperature and the incidence of HFMD remains unclear in Chongqing. In this study, we aimed to quantitatively evaluate the effect of daily mean temperature on the incidence of HFMD in children in this area, and in turn, provided a scientific basis for the prevention and control of HFMD.

## Materials and methods

### Study area

Chongqing is located in the southwestern of China with the latitude (28° 10′–32° 13′ N, 105° 11′–110° 11′ E), which features a humid subtropical monsoon climate, with an annual daily mean temperature of 16–18 °C and relative humidity of 70–80%.

### Data

The daily cases of HFMD in Chongqing from January 1, 2012 to December 31, 2019 were collected from the Notifiable Infectious Disease Surveillance System, Chinese Center for Disease Control and Prevention (CISDCP). HFMD was classified as a class “C” disease by the Ministry of Health of China, which demands that all HFMD cases should be reported to CISDCP within 24 h after diagnosis. The clinical criteria for diagnosis of HFMD cases were provided in a guidebook published by the Chinese Ministry of Health (http://www.nhc.gov.cn/).

The daily meteorological data, including daily mean temperature, atmospheric pressure, relative humidity, duration of sunshine and wind speed, were obtained from the China Meteorological Bureau (http://data.cma.cn/).

### Statistical analysis

We used distributed lag non-linear models (DLNMs) to assess the associations between the daily mean temperature and HFMD cases. DLNM is a flexible model that estimates the nonlinear and delayed effects of exposure–response relationship between a climatic factor and disease^26,27^. Spearman’s correlation was used to examine the relevance between the daily meteorological data and the incidence of HFMD. The time-series distribution of meteorological factors and the incidence of HFMD were explored by scatter plots. The variance inflation factor (VIF) was applied to assess the co-linearity. If the VIF exceeds 5, it would indicate multicollinearity^28^. Moreover, except for daily mean temperature, other meteorological factors related to the incidence of HFMD were included in the model as cofounders.

To avoid over-dispersion of the HFMD, Poisson regression was constructed with a quasi-Poisson function that allows for over-dispersion in the daily HFMD cases to combine DLNMs. The model was described as following:

$$\begin{aligned} \log \left[ {E\left( {Y_{t} } \right)} \right] & = \alpha + cb\left( {Temp_{t} ,lag} \right) + \sum ns\left( {weather_{t} ,df = 3} \right) \\ & \quad + ns\left( {time,3*8} \right) + DOW + holiday + periodicity \\ \end{aligned}$$

where $E\left( {Y_{t} } \right)$ was the number of daily HFMD cases on day t; $\alpha$ was the intercept; *cb* denoted the cross-basis function of temperature and lag time; *ns*() indicated a smooth function based on natural cubic spline; *df* was the degree of freedom; *time* refereed seasonality and the long-term trend, and we used 8*df* per year for controlling seasonality and long-term trend; *DOW* refereed the day of the week; *holiday* was a binary variable for public holidays; *periodicity* was a binary variable of high incidence year. Referring to previous studies, the degree of freedom of other meteorological factors was set to 3^27,29,30^; According to the median incubation period of HFMD and previous research results, the longest lag time was determined to be 21 days^23,31–33^. We used the Akaike information criterion for quasi-Poisson (Q-AIC) to choose the degree of freedom for the meteorological variables and the *df* for their lags^31^. The degree of freedom of the cross-base in the temperature and time is generally between 2 and 5. The model was established by using the combination of the parameters, and the lowest point of the effect was taken as the reference.

The analysis process was divided into two stages. Firstly, DLNMs were used to establish the dose–response relationships between daily mean temperature and HFMD incidences of children < 5 years old in total. Then, stratified analyses were conducted by gender, different age groups, and children categories.

All statistical tests were two-sided, and *p* < 0.05 was considered statistically significant. Rstudio 5.4.1 software was used for descriptive analysis and Spearman rank correlation analysis. The DLNM model was fitted and visualized by mgcv, splines, dlnm, and ggplot2 packages in Rstudio 5.4.1 software. The temperature (8 °C) corresponding to the minimum effect was used as a reference to calculate the RR value.

Sensitivity analyses were performed for the data by changing the degree of freedom of meteorological factors, *df* (6, 7, 8) for time to control for seasonality and secular trend, and maximum lag days (14, 21, 28) for daily mean temperature.

### Ethics approval and consent to participate

Protocol for each study conducted using the collected data was submitted to the Ethics Committee of the Chongqing Center for Disease Control and Prevention, which ensures that the use of data falls within the scope specifically agreed upon by hand, foot, and mouth patients.

## Results

### Descriptive analysis

A total of 413,476 HFMD cases aged < 5 years were reported in Chongqing from January 1, 2012 to December 31, 2019. The boy-to-girl ratio was 1.39:1, and majority of the cases were aged 1–2 years (57.38%). Almost 70% of the HFMD cases were scattered children, while the others were daycare children.

**Figure 1:**
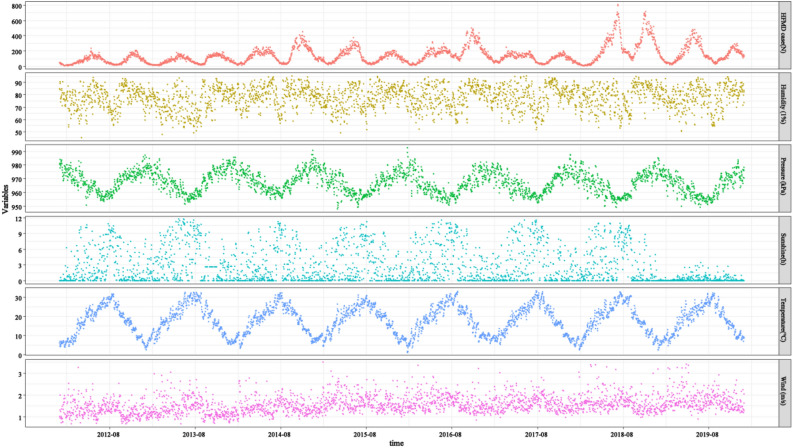
The time-series distribution of daily HFMD case, mean relative humidity, sunshine hours, mean pressure, daily mean temperature, and mean wind speed.

The descriptive statistics of meteorological factors from 2012 to 2019 were presented in Table 1. During the study period, the daily mean temperature ranged from 1.14 to 33.51 °C with the mean of 17.87 °C. The daily relative humidity, sunshine hours, atmospheric pressure, and wind speed were ranged from 45.17 to 95.27%, 0 to 11.73 h, 94.79 0.1 to 99.26 0.1 hPa and 0.67 to 3.49 m/s, respectively (Fig. 1, Table 1).

**Table 1:** Descriptive statistics of meteorological factors.

Meteorological factors	Percentile			Min	Max	Mean (SD)
	25th	50th	75th			
Relative humidity (%)	71	78.83	84.92	45.17	95.27	77.60 (9.36)
Sunshine (h)	0.03	0.96	4.67	0	11.72	2.68 (3.33)
Atmospheric pressure (0.1 hPa)	96.04	96.71	97.33	94.79	99.26	96.71(7.97)
daily mean temperature (°C)	10.75	18.16	24.04	1.14	33.51	17.87 (7.66)
Wind speed (m/s)	1.28	1.5	1.79	0.67	3.49	1.56 (0.41)

*SD* standard deviation, *Max* maximum, *Min* minimum.

Figure 1 showed the time-series distribution of daily HFMD cases, relative humidity, sunshine hours, atmospheric pressure, daily mean temperature, and wind speed in Chongqing from 2012 to 2019. HFMD cases were reported throughout the year with semiannual peaks, which were from April to July and from October to December each year.

Spearman’s correlations between daily meteorological variables and HFMD cases were shown in Table 2. There were nonlinear relationships between meteorological factors and HFMD incidence, except for sunshine hours and wind speed. The relative humidity and daily mean temperature were positively correlated with the incidence of HFMD. In contrast, atmospheric pressure was negatively related to HFMD incidence, with a strong correlation (r = − 0.839, *p* < 0.01).

**Table 2:** Spearman’s correlations between meteorological variables and HFMD cases in Chongqing, 2012–2019.

Variables	HFMD cases	Relative humidity	Sunshine	Atmospheric pressure	Daily mean temperature	Wind speed
HFMD cases	1					
Relative humidity	0.188*	1				
Sunshine	− 0.028	− 0.639*	1			
Atmospheric pressure	− 0.119*	0.215*	− 0.334*	1		
Daily mean temperature	0.141*	− 0.313*	0.509*	− 0.839*	1	
Wind speed	0.026	− 0.287*	0.083*	− 0.341*	0.23*	1

*p < 0.05.

### Dose–response relationship between daily temperature at different lag times and HFMD cases

According to the principle of the smallest of Q-AIC, the degrees of freedom of the temperature, lag time and degrees of freedom of time in the cross base were determined to be 5, 4 and 8, respectively. The meteorological variables finally included in the model for control included relative humidity, sunshine hours, pressure, and wind speed.

**Figure 2:**
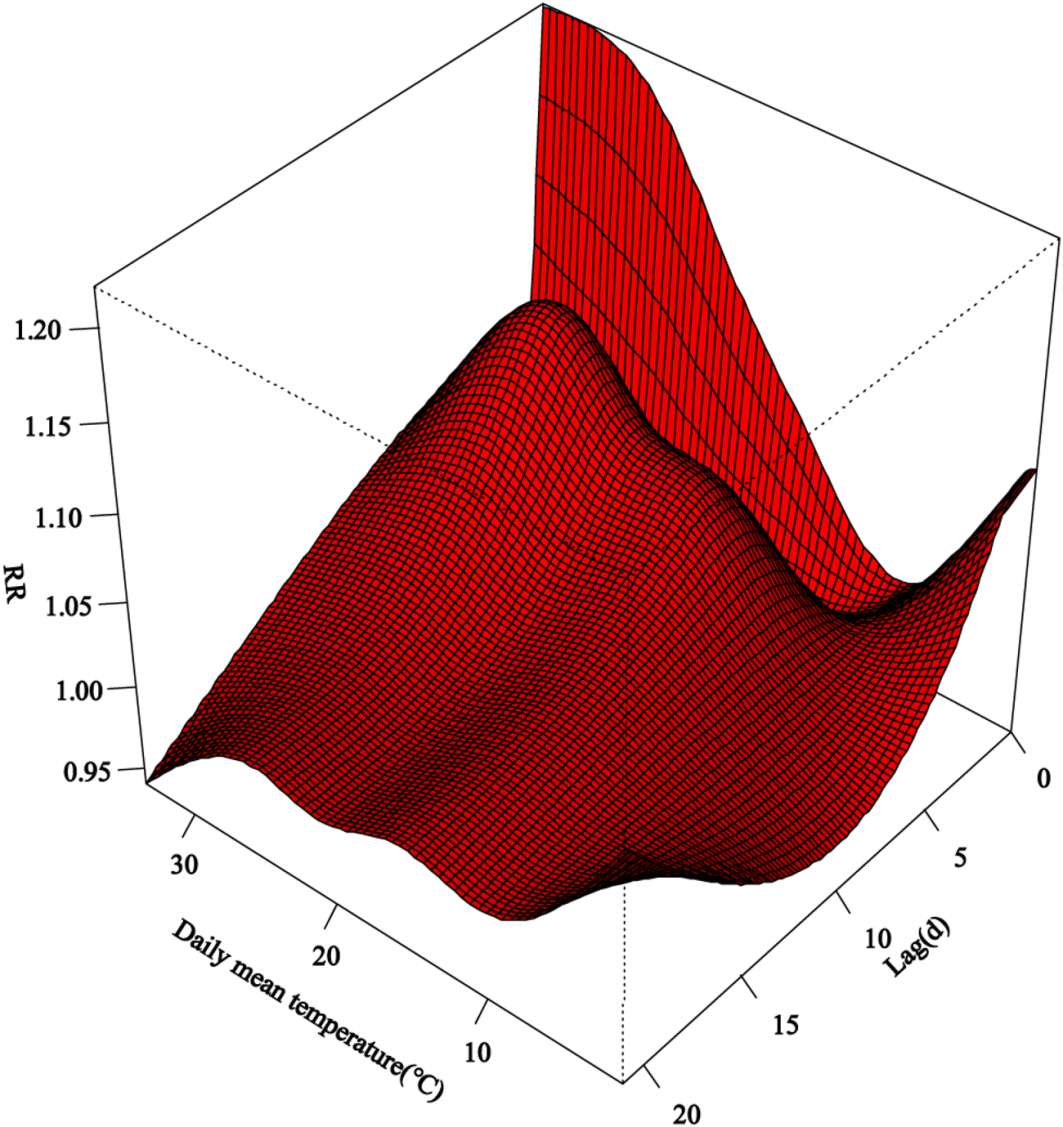
Three-dimensional plot for HFMD along lags with reference at 8 °C by DLNM models in Chongqing.

Taking the temperature (8 °C) corresponding to the minimum effect as the reference, the daily mean temperature had a non-linear relationship with the daily HFMD cases at different lag times. Figure 2 showed that the risk was the highest when the daily mean temperature was 26.24 °C at lag 5 days (RR = 1.12, 95% CI 1.10–1.14). While the risk was the lowest when the daily mean temperature was 33.51 °C at lag 21 days (RR = 0.94, 95% CI 0.91–0.97).

**Figure 3:**
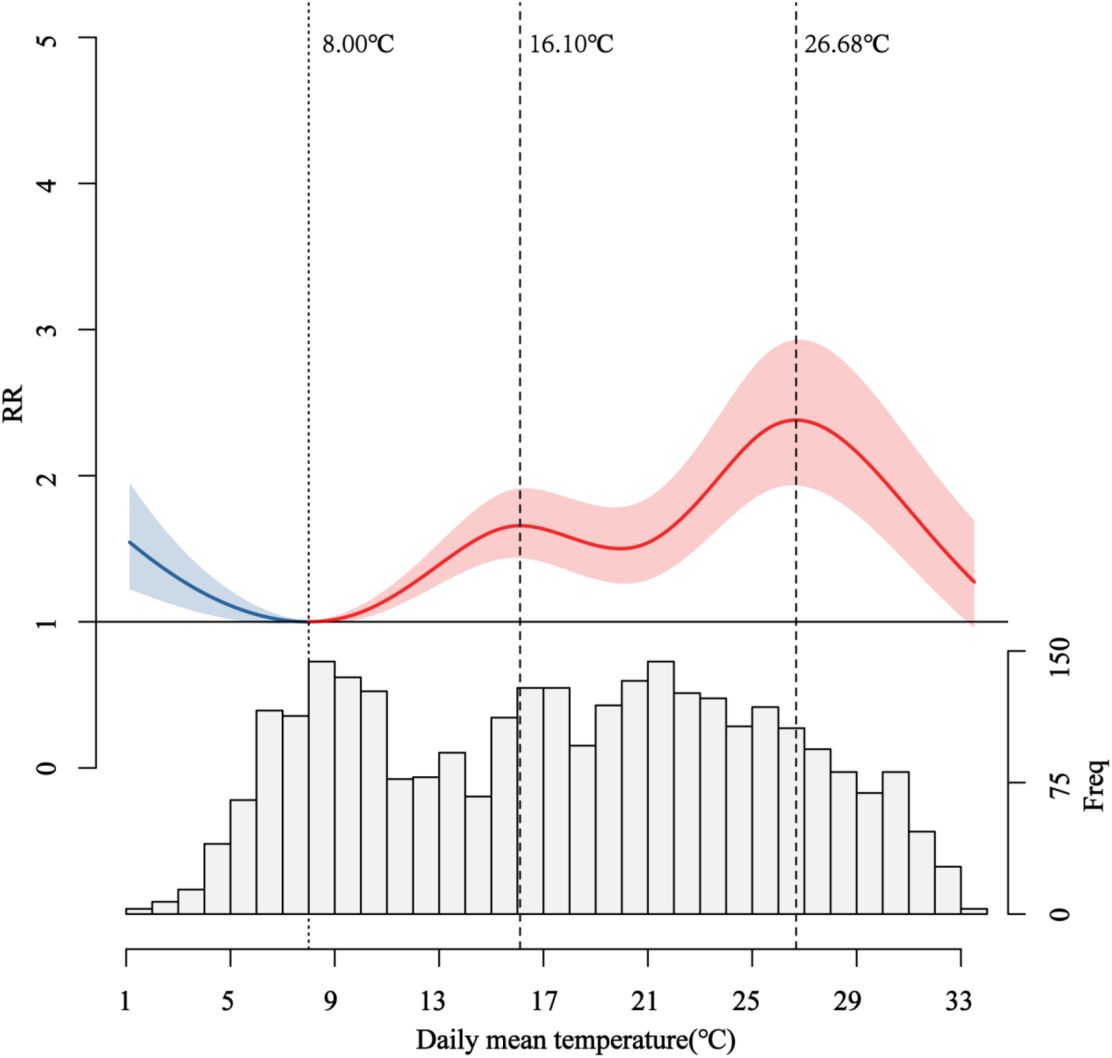
Overall effects of daily mean temperature on HFMD in Chongqing. The blue line represented the temperature below the effect minimum temperature, and the red line represented the temperature at or above the effect minimum temperature. Vertical dotted lines represented local minima and maxima.

Figure 3 showed the overall cumulative dose–response relationship between daily mean temperature and HFMD cases at lag 21 days. The relationship between daily mean temperature and HFMD could be described as a wavy-shaped curve. Using 8 °C as a reference, statistical significance was observed when the daily mean temperature was below 5.66 °C or above 9.43 °C. If the daily mean temperature was between 5.66 and 9.43 °C, no statistical significance was observed. When the daily mean temperature was below 5.66 °C, the RRs value increased with decreasing temperature. Conversely, when the daily mean temperature was above 9.43 °C, the RRs value increases with increasing temperature. The RRs increased as the daily mean temperature was below 5.66 °C or above 9.43 °C, with two peaks at 16.10 °C (RR = 1.65, 95% CI 1.44–1.91) and 26.68 °C (RR = 2.38, 95% CI 1.93–2.93).

### Relationships between daily mean temperature and HFMD cases at different lags

**Figure 4:**
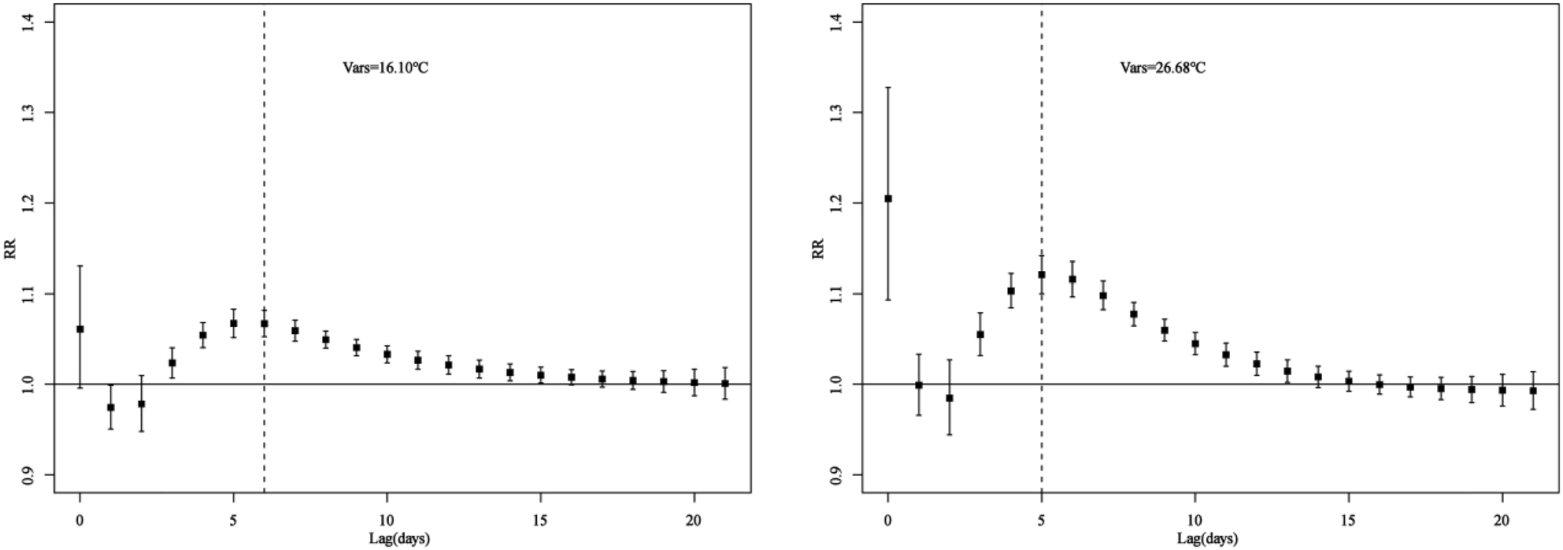
The relative risks of different daily mean temperatures for HFMD cases at different lags. The temperature 8 °C was defined as the reference for calculating relative risk. The temperature 16.10 °C and 26.68 °C represented the daily mean temperature and highest RR temperature, respectively.

Figure 4 showed the effect of different daily mean temperature (16.10 °C, 26.68 °C) on HFMD cases compared with 8 °C, at different lag days. The lag effects of daily mean temperature of 16.10 °C and 26.68 °C on HFMD were similar, which showed a rapid increase RR at the current day, followed by lag 1 day (the previous day) and lag 2 days (the previous two days), then exhibited an inverted V-shape until lag 21 days. The RRs reached the highest at the current day when the daily mean temperature was 26.68 °C (RR = 1.20, 95% CI 1.09–1.32) and at lag 5 days when the daily mean temperature was 16.10 °C (RR = 1.07, 95% CI 1.05–1.08).

### Cumulative RRs of daily mean temperature on HFMD cases stratified by gender and children category

**Figure 5:**
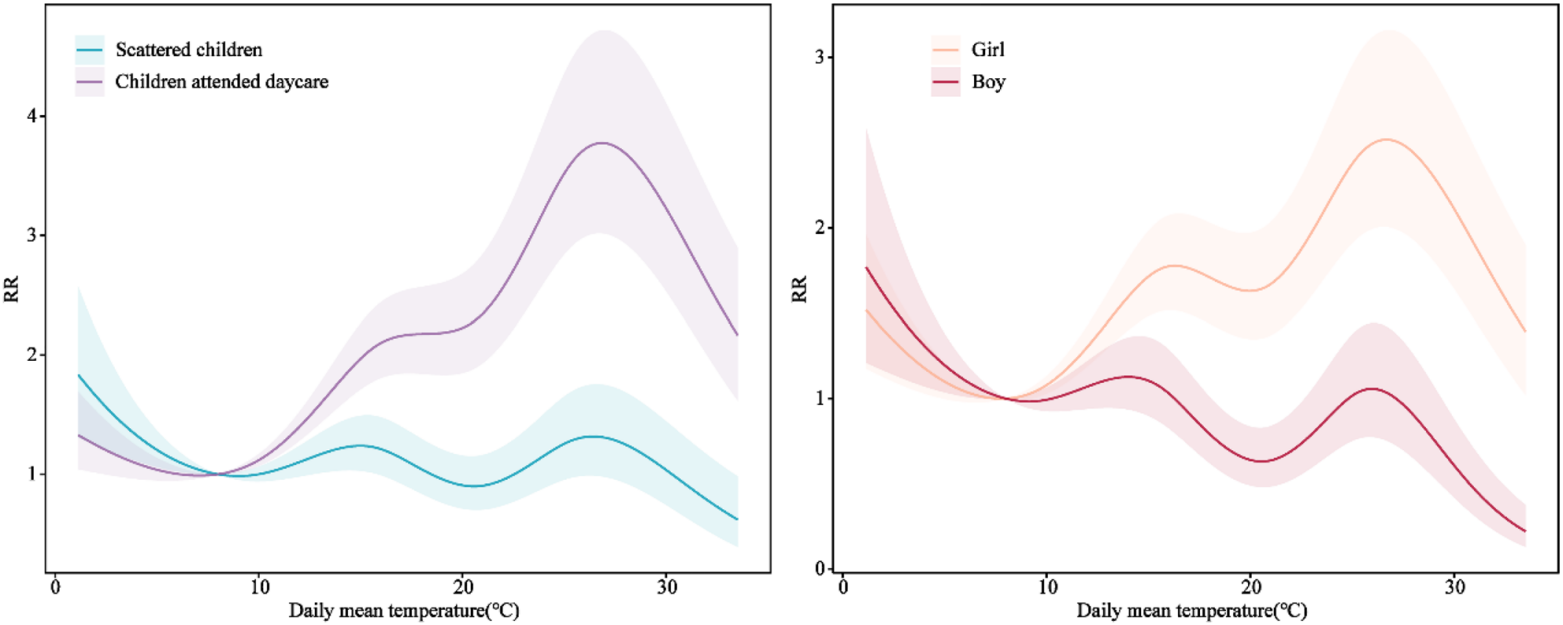
The relative risks of different temperatures for HFMD cases stratified by children category and gender. The temperature 8°Cwas defined as the reference for calculating relative risks.

Figure 5 illustrated the cumulative RRs of daily mean temperature on HFMD cases stratified by gender and children category. In general, the RRs of daycare children and girls were much higher than scattered children and boys. The RR of daycare children reached the highest (RR = 3.78, 95% CI 3.02–4.72) at 26.84 °C, while the RR of girls reached the highest (RR = 2.52, 95% CI 2.01–3.16) at 26.68 °C, respectively.

## Discussion

In this study, we examined the effect of daily mean temperature on the incidence of HFMD in children under 5 years old in Chongqing, southwest of China. To our knowledge, this is the first study to report the relationship between daily mean temperature and the incidence of HFMD in this area.

In our study, we found the relationship between daily mean temperature and HFMD was illustrated with a wavy-shaped curve, which differs from previous studies. Onozuka et al. and Kim et al. reported an inverted V-shaped relationship between the weekly temperature and HFMD in Japan and South Korea^12,23^, while Hii et al. showed a J-shaped relationship in Singapore^24^. Conversely, Yin et al. and Huang et al. indicated a M-shaped or an inverted V-shaped relationships between the daily temperature and HFMD in Chengdu and Wuhan, respectively^34,35^. The variability in these findings can be partly attributed to differences in methodologies and data sources, suggesting that the temperature-HFMD relationship might be influenced by location-specific variables. The reasons for the differences need further investigation.

The RRs of a dose–response relationship between daily mean temperature and HFMD cases showed two peaks at 16.10 °C and 26.68 °C in Chongqing. Temperature influences the survival and spread of infectious pathogens in the environment, as well as the behaviors and activities of the population, thereby influencing the dynamics of infection transmission^36,37^. During warmer periods, people are more likely to take outside activities rather than stay at home, which may contribute to the spread of enteroviruses through respiratory droplets, ruptured skin vesicles, or direct contact with contaminated toys and environmental surfaces, all of which increase the frequency of contact, and therefore the occurrence of HFMD.

Regarding the lag times, the RRs of 16.10 °C and 26.68 °C on the incidence of HFMD reached the highest at lag 5 days and at the current day, respectively. Higher temperature (26.68 °C) had a shorter lag effect on HFMD than lower temperature (16.10 °C). Our results aligned with the earlier reported hypothesis that low-temperature associated risks occurred at slower rates and lasted shorter while high-temperature associated risks occurred at faster rates and lasted longer^38^. The finding may be related to the fact that temperatures can have influence on the development and longevity of the virus^39^. For instance, the virus could reproduce more rapidly and survive for longer times at high temperatures than at low temperatures, so the occurrence of HFMD cases was immediate and lasted longer days at high temperature. Meanwhile, the period of high temperature in summer is the season of a high incidence of intestinal infectious diseases. The HFMD cases in this period may be treated in time due to strong awareness of infectious disease protection. Moreover, the lag of treatment may also affect the lag effect of temperature on disease epidemic, which needs further research to verify.

In subgroup analysis, the influence of temperature on the incidence of HFMD varied between different genders and children categories, which were consistent with previous studies^13,35,39,40^. Children who attended daycare were more sensitive to temperature change, which might be due to more frequent activities with other children in a confined environment, such as sharing toys and beds^19^. We found that the effects of temperature on HFMD for girls were higher than that for boys, which was consistent with the result in the study by Cheng et al.^17^, but inconsistent with the results of studies by Huang and Koh et al.^41,42^. The differences between boys and girls might be due to the different socioeconomic characteristics of families and adaptive capacity of children at different locations^38^. Therefore, it is crucial to take preventive measures before the epidemic season such as strengthening health promotion, vaccination, etc. which could reduce the incidences of HFMD.

This study has several limitations. First, the data on HFMD were collected through passive monitoring; therefore, the actual numbers of HFMD cases may have been underestimated. Second, this study was conducted in Chongqing. The results may not be applicable in other areas, especially regions with different weather patterns. Third, the study was an ecological research at the population level rather than the individual level, and misclassification bias was inevitable. Last, the meteorological data were taken from fixed monitoring sites rather than individual exposure measures, which may create measurement errors in the exposure. However, these errors were likely to be random.

In conclusion, the study demonstrated a non-linear association between daily mean temperature in Chongqing. The high-temperature risk was greater than the low-temperature. Girls and daycare children were more vulnerable to the temperature, which could be used to improve early warning systems and preventive measures for HFMD.

## Data Availability

The datasets used and analyzed during the current study were collected from the Notifiable Infectious Disease Surveillance System, Chinese Center for Disease Control and Prevention and China Meteorological Bureau, but the availability of these data was limited, so they were not disclosed. However, data can be obtained from the corresponding author Li Qi (E-mail: cfs@cqcdc.org) on reasonable request.
